# The SARS-CoV-2 nucleocapsid protein: its role in the viral life cycle, structure and functions, and use as a potential target in the development of vaccines and diagnostics

**DOI:** 10.1186/s12985-023-01968-6

**Published:** 2023-01-10

**Authors:** Wenbing Wu, Ying Cheng, Hong Zhou, Changzhen Sun, Shujun Zhang

**Affiliations:** 1 Department of Biochemistry and Molecular Biology, School of Basic Medical Sciences, Southwest Medical University, Luzhou, 646000 China; 2 Drug Research Center of Integrated Traditional Chinese and Western Medicine, The Affiliated Traditional Chinese Medicine Hospital, Southwest Medical University, Luzhou, 646000 China

**Keywords:** SARS-CoV-2, COVID-19, Nucleocapsid protein, Post-translational modifications, Liquid–liquid phase separation, Diagnostics, Vaccines

## Abstract

Coronavirus disease 2019 (COVID-19) continues to take a heavy toll on personal health, healthcare systems, and economies around the globe. Scientists are expending tremendous effort to develop diagnostic technologies for detecting positive infections within the shortest possible time, and vaccines and drugs specifically for the prevention and treatment of COVID-19 disease. At the same time, emerging novel variants have raised serious concerns about vaccine efficacy. The SARS-CoV-2 nucleocapsid (N) protein plays an important role in the coronavirus life cycle, and participates in various vital activities after virus invasion. It has attracted a large amount of attention for vaccine and drug development. Here, we summarize the latest research of the N protein, including its role in the SARS-CoV-2 life cycle, structure and function, and post-translational modifications in addition to its involvement in liquid–liquid phase separation (LLPS) and use as a basis for the development of vaccines and diagnostic techniques.

## Introduction

The SARS-CoV-2 global pandemic has had a significant impact on public health and economies around the world [1, 2]. Since the end of 2020, a variety of mutations of the SARS-CoV-2 strain have appeared, which can potentially seriously threaten the health of human beings and impose significant long-term impacts on human production and living activities [3–12]. Thus, vaccines and antiviral drugs against SARS-CoV-2 are urgently needed. The current strategy for addressing SARS-CoV-2 are aimed at attacking the main link in the life cycle of the virus [6, 13–16]. It is therefore becomes imperative to understand the life cycle of SARS-CoV-2 at the molecular level and to analyze the structure and function of its constituent proteins [17]. Among the four structural proteins and sixteen nonstructural proteins encoded by the SARS-CoV-2 genome, the majority of attention has been focused on the coronavirus spike (S) proteins due to their importance in the viral life cycle [17, 18]. However, many other SARS-CoV-2 proteins play an equally important role in the virus life cycle, but we know relatively little about their structures or biophysical properties [19, 20]. Among them, the N protein is a particularly attractive antiviral target. In fact, the N protein is not only the basis of viral RNA genome packaging into ribonucleotide complex (RNP) and assembly into virus particles but also is the most abundant protein in virions and a high immunogenicity antigen. Moreover, it is also the determinant of virulence and pathogenesis [17, 21–23]. The N protein can not only be used as a potential target for therapy or vaccines but also as an important diagnostic marker for COVID-19 [20, 24, 25]. Therefore, this paper focuses on the SARS-CoV-2 life cycle, the structure and function of N protein domains, the post-translational modification of the N protein, the mechanism and function of N protein-related LLPS, and the N protein-based development of vaccines and diagnostics. This information may help in designing effective drugs and vaccines to end the SARS-CoV-2 pandemic.

### SARS-CoV-2

**Fig. 1:**
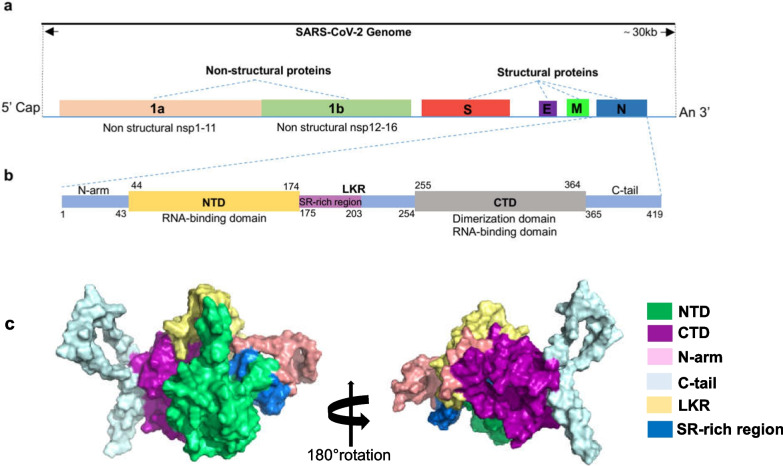
Genome organization of SARS-CoV-2 and structural overview of the SARS-CoV-2 nucleocapsid protein.** a**. Genome organization of SARS-CoV-2. **b**. Schematic representation of SARS-CoV-2 N protein domains. The N-terminal domain (NTD), C-terminal domain (CTD) and three intrinsically disordered regions (IDRs), i.e. N-arm, linker region (LKR) and C-tail are illustrated. The charged Ser/Arg (SR)-rich region is shown. **c**. Deletion analyses of N protein of SARS‐CoV‐2. Structures were visualized in PyMol v2.4

SARS-CoV-2 is a pleomorphic particle with a diameter of approximately 60–100 nm [26, 27]. The length of its RNA genome is approximately 30 kb, and the specific structural composition is shown in Fig. 1a and b: it contains two large overlapping open reading frames, ORF1a and ORF1b, which are further processed to produce 16 nonstructural proteins (Nsp1 to 16) [28]. Additionally, it encodes 4 structural proteins, namely the spike (S), membrane (M), envelope (E), and nucleocapsid (N) proteins and 9 auxiliary proteins (ORF3a, ORF3b, ORF6, ORF7a, ORF7b, ORF8, ORF9b, ORF9c, and ORF10) [9, 28–32]. The structures of some of these proteins have been resolved, and their functions have been studied in depth [31, 33–38].

### The role of the N protein in SARS-CoV-2 life cycle

**Fig. 2:**
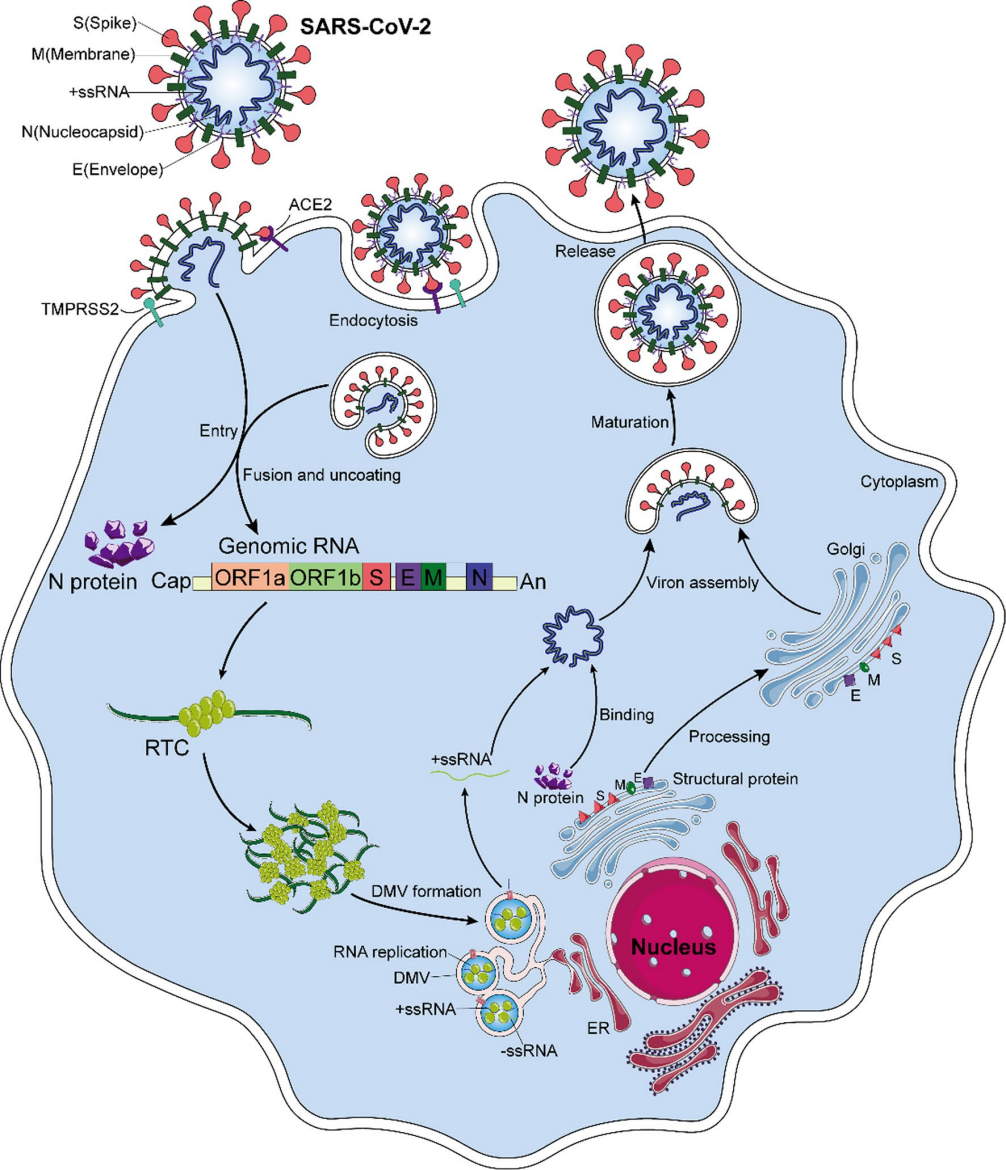
Depicts the SARS-CoV-2 life cycle. When the SARS-CoV-2 spike protein binds to the target cell receptor ACE2, the S protein is cleaved by host cell proteases, such as TMPRSS2, triggering fusion of the virus with the plasma membrane. In addition, SARS-CoV-2 can enter cells through endocytosis. The N protein is the dissociated from the positive strand (+) RNA genome of SARS-CoV-2 and translated into polyproteins pp1a and pp1ab. These polyproteins are translated and processed into nonstructural proteins nsp1-16, which build viral replication and transcription complexes (RTCs) and reshape the cell membrane to form replicating organelles (DMVs). These organelles form a continuum with the endoplasmic reticulum, and viral RNA replication mainly occurs in DMVs. The newborn virus RNA exits DMVs through transmembrane pores to reach the location for translation or virion assembly. The translated structural proteins are transported to the endoplasmic reticulum (ER) membrane and transit through the ER-to-Golgi intermediate compartment (ERGIC). The positive strand RNA of the genome wrapped by the N protein undergoes assembly with structural proteins S, M, and E, and new virions are formed by budding into the lumen at ERGIC. Finally, the progeny virions are released from the host cell

The life cycle of the SARS-CoV-2 is shown in Fig. 2 [26, 39]. The initial stage of coronavirus infection involves the specific binding of the SARS-CoV-2 spike (S) protein to host cell entry receptors [26, 39–41]. The spike protein, which is a homotrimeric class I fusion glycoprotein, consists of S1 and S2 subunits with different functions. The surface-exposed S1 subunit receptor binding domain (RBD) specifically binds to the host cell receptor angiotensin-converting enzyme 2 (ACE2) [37, 42–46]. The transmembrane S2 domain comprises heptapeptide repeat regions and fusion peptides. When hydrolyzed by host cell-derived serine protease TMPRSS2, they mediate fusion of the virus and host cell membrane through extensive conformational rearrangement [39, 47, 48]. Host cysteine protease cathepsin B (CatB) and CATL facilitate this process [39, 47, 50–52]. In addition, a multi-alkali cleavage site (PRRAR) in the prototype protein converting enzyme Furin can be found at the boundary of S1–S2, and Furin cleavage leads to increased infection [46, 53–55]. In addition, SARS-CoV-2 can enter the cells through endocytosis, and fusion is then induced by S cleavage of endosomal/lysosomal proteins at low Ph [26, 39]. When SARS-CoV-2 enters the host cell, the N protein is dissociated from the positive strand (+) RNA genome of the virus, and the viral gene replication and expression program begins, which is highly regulated in space and time [26]. In host cells, there is an inherent antiviral immune defense mechanism, called RNA interference (RNAi), which can lead to degradation of the virus genome to inhibit virus replication [56–58]. The N protein acts as a viral inhibitor of RNAi in host cells [56, 57]. In the initial step, the dsRNA in infected cells can be intercepted by the N protein, thus preventing the recognition and cleavage of viral dsRNA [57, 58]. On the one hand, the positive strand (+) RNA genome of the virus translates ORF1a and ORF1b into polyprotein pp1a and pp1ab, respectively [26, 39, 59]. After the cleavage of two cysteine proteases, nsp3 (papain-like protease, PL^pro^) and nsp5 (chymotrypsin-like protease, also known as 3C protease 3CL^pro^, or major proteolytic enzyme, M^pro^), nonstructural proteins are produced [26, 39, 59, 60]. NSP1 shuts down host translation and promotes host mRNA degradation. Nsp2-11 regulates the intracellular environment to facilitate viral replication. Nsp12-16 contains core enzymes needed for RNA synthesis, including RNA-dependent RNA polymerase (RdRp; NSP 12), while NSP2-16 and N protein establish a viral replication-transcriptional complex (RTC) and reshape the cell membrane to form replicating organelles, which are essential for keeping RNA replicated and transcribed in an orderly conformation [26, 42, 61, 62]. These organelles are connected to the endoplasmic reticulum (ER), providing the best environment for viral RNA replication [26, 39]. On the other hand, viral RNAs are replicated in double-membrane vesicles (DMVs) [26, 39]. Replication begins with the synthesis of negative-strand RNA copies, which are used as templates to synthesize new plus-strand RNA genomes that may enter additional rounds of translation or be incorporated into new virions. The discontinuous transcription of positive-strand genomic RNA produces subgenomic minus-strand RNA, which is used as a template to synthesize subgenomic positive-strand RNA that encodes structural proteins and helper proteins [26, 39]. The newborn virus RNA exits DMVs via transmembrane pores to reach the location for translation or virion assembly [26]. Subsequently, the positive-strand RNA of the genome is encapsulated with the N protein and undergoes assembly with structural proteins S, M, and E in the ER–Golgi intermediate compartment (ERGIC), and new virions are formed by budding into the lumen at ERGIC. Finally, the offspring virions may be released from the host cells via exocytosis [26]. However, recent evidence suggests that SARS-CoV-2 is more likely to exit infected cells through lysosomal transport [39, 63]. In general, N proteins are responsible for regulating host cell cycle progression, host–pathogen interactions, and apoptosis [64–67]. In addition, the N protein has strong immunogenicity, can induce protective immune responses, and is highly expressed during infection [20, 68]. The exploitation of host cellular mechanisms plays a regulatory role in the virus life cycle and is the key to the integration of viral RNA into virus offspring particles. Thus, the N protein plays an important role in the virus infecting host cells.

### Structure and function of the N protein

The N protein is a structurally heterogeneous, 419-amino acid-long, multi-domain RNA-binding protein (Fig. 1b and c) [69]. Like other coronaviruses, the N protein also has two conserved, independently folded domains, namely the N-terminal domain (NTD) and the C-terminal domain (CTD) (Fig. 1b) [21, 28]. These two domains are connected by an inherently disordered region (IDR) called the central linking region (LKR). The LKR includes a Ser/Arg (SR)-rich region, which contains putative phosphorylation sites [26, 28, 39, 70]. In addition, there are two IDRs on both sides of the NTD and CTD, which are called N-arm and C-tail. NTD is responsible for RNA binding, CTD is responsible for RNA binding and dimerization, and IDR is responsible for regulating the RNA-binding activity and oligomerization of NTD and CTD [21, 28]. Next, this paper will present the latest research progress on characterizing the structure and function of N protein domains.

### Structure and function of NTD

**Fig. 3:**
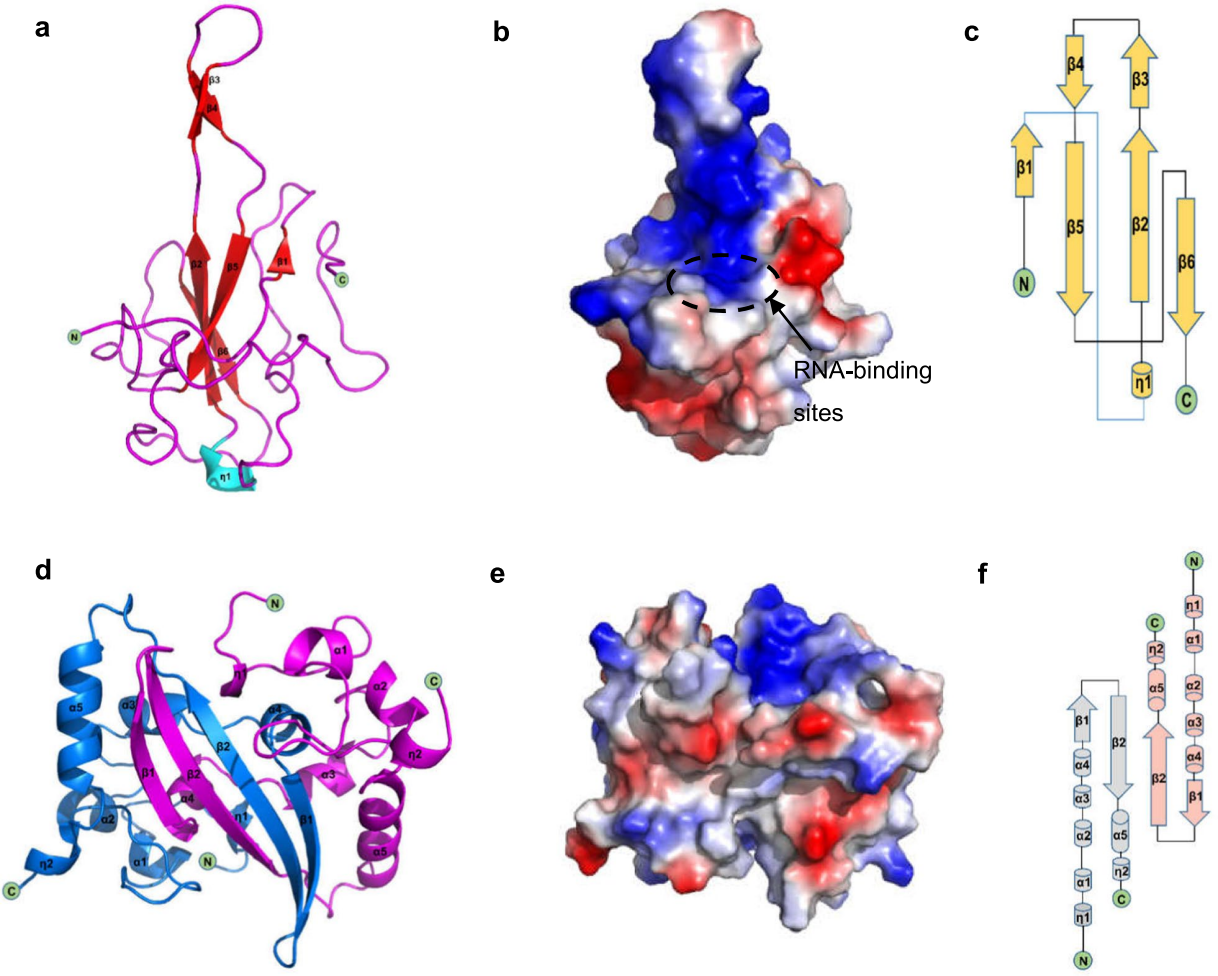
Structures of SARS-CoV-2 NTD and CTD. **a** Cartoon representation of SARS-CoV-2 NTD. **b** The electrostatic surface potential of SARS-CoV-2 NTD. Red and blue colours indicate negative and positive potential, respectively. The RNA-binding sites are highlighted in dotted circles and labelled. **c** Topology diagram for SARS-CoV-2 NTD; *β* represents the* β*-sheet and η represents the 3_10_ helix. **d** Cartoon representation of SARS-CoV-2 CTD. **e** The electrostatic surface potential of SARS-CoV-2 CTD. Red and blue colours indicate negative and positive potential, respectively. **f** Topology diagram for SARS-CoV-2 CTD; α represents the *α*-helices, *β* represents the *β*-sheet and *η* represents the 3_10_ helix

A number of scientific teams have successfully resolved the structure of SARS-CoV-2 NTD [28, 38, 71, 72]. Its structure is very similar to the N proteins of other coronaviruses [28, 71, 72]. The SARS-CoV-2 NTD takes the shape of a right-handed fist (Fig. 3 a). It consists of a four-strand antiparallel *β*-fold core subdomain, which is located between the annular or short 3_10_ helix and the prominent *β*-hairpin region formed by *β*2 and *β*3 chains outside the nucleus(Fig. 3c). There is a large protruding *β*-hairpin between *β*2 and *β*3 that acts as a bridge connecting them and protrudes from the core (PDBID:6YI3 and 7CDZ); the *β*-hairpin has a high degree of flexibility [28, 71, 72]. The N protein has the ability to recognize and bind RNA [28, 72, 73]. In NTD, the prominent *β*-hairpin is mainly composed of basic amino acid residues. Further analysis of the surface electrostatic potential shows that there is a positively charged pocket at the connection between the basic hairpin and the core structure that is considered to be an RNA-binding site, and this site is conserved between different coronaviruses that infect humans (Fig. 3b) [71]. By constructing an atomic model of the protein–RNA complex, Dinesh et al. [71] demonstrated that both dsRNA and ssRNA bind to the positively charged canyon between the alkaline *β*-hairpin and the core of NTD in a similar way, and the arginine residues R92, R107, and R149 that directly bind RNA are located in the canyon [28, 71, 72].

Although the overall structure of SARS-CoV-2 NTD is very similar to that of other coronavirus NTD structures, there are significant structural differences in several regions. First, compared with all other existing CoV NTD structures that cause human infections, the N-terminal loop of SARS-CoV-2 extends outward, while the circumferential core subdomain of HCoV-OC43 and HCoV-NL63 NTD rotates [28, 38, 69]. Second, the *β*-hairpin region protruding from SARS-CoV-2 NTD is flexible, but the flexible ring cannot even be seen in HCoV-OC43 or HCoV-NL63 NTD structures. Additionally, the residues that bind to AMP are also different. The N-terminal region of the SARS-CoV-2, SARS-CoV, and MERS-CoV NTDs is similar to that of HCoV-OC43 and contains conserved residues. The phenolic hydroxyl of Y124 interacts with the adenine ring of AMP through a hydrogen bond, while the skeleton of G68 forms a hydrogen bond with the monophosphate group of AMP [28, 71, 72]. For HCoV-NL63, residues H77 and P24 lack the ability to form hydrogen bonds, which may further block the binding of AMP. Additionally, in different coronavirus NTD structures, the electrostatic surface potential also shows different charge distribution patterns, especially corresponding to the N-terminal ring, the top of the protruding region, and the bottom of the core subdomain [28]. In addition, the N-terminal tail residues (Asn48, Asn49, Thr50, and Ala51) of SARS-CoV-2 NTD are more flexible and extend outward to a greater extent than their equivalent residues in HCoV-OC43 NTD, and this may facilitate opening of the binding pocket to adequately fit the high-order structure of the viral RNA genome [38].

### Structure and function of N-CTD

Similarly, the SARS-CoV-2 CTD crystal structure has been characterized by several research teams [17, 28, 72, 74]. Usually, two CTD monomers combine to form dimers (Fig. 3d) (PDB:6YUN,7CE0 and 6ZCO), which are diamond-shaped and tile-shaped, and each monomer consists of five *α*-helices, two 3_10_-helices, and two *β*-strands (Fig. 3f) [17]. The *β*-hairpin from one prototype is inserted into the cavity of another prototype, resulting in the formation of a four-chain, antiparallel *β*-strand at the dimer interface. The *β*-strand forms one side of the dimer, while on the other side of the dimer, the surface is formed by an *α*-helix and a ring. The extensive hydrogen bonding interaction between the two hairpins and the hydrophobic interaction between the *β*-sheet and α-helix result in the dimer structure being highly stable [17, 28]. The CTD structure of SARS-CoV-2 is highly similar to that of SARS-CoV, MERS-CoV, and HCoV-NL63. All of them show a conservative positively charged groove on the helicoid of the N-CTD dimer, and it is speculated that they contain RNA-binding sites [17, 28, 72, 73, 75, 76]. In SARS-CoV CTD, the hypothetical RNA-binding site is located at 248–280 aa and is conserved [17, 75, 76]. The residues identified as RNA-binding sites in SARS-CoV2 CTD correspond to Arg319, Thr334, and Ala336 [34, 76]. The analysis of the electrostatic surface potential of these positions shows that the positively charged residues belonging to these amino acid regions gather at the relative edge of the basic groove, extending laterally relative to the dimer interface line (Fig. 3e) [17, 28]. The positive charge in this region of SARS-CoV-2 CTD is mainly due to the distribution of several positively charged residues, including K256, K257, K261, and R262 [28, 72]. However, the electrostatic potential surfaces of the *β*-strand surfaces of these proteins show different characteristics [28]. The MERS-CoV structure shows a positively charged central region, while the SARS-CoV-2 and SARS-CoV structures both show a negatively charged region. For HCoV-NL63, the central region of its *β*-folding surface shows a highly negatively charged region. These differences may affect the binding pattern of RNA recognition [28]. According to the proposed model of the coronavirus RNP complex, CTD primordia are packaged into a spiral core, around which the ssRNA of the genome is distorted, so each CTD contains a single-stranded RNA channel consisting of seven bases in its positively charged groove [17, 64]. Microscale thermophoresis demonstrated that binding occurs to a 7-nucleotide fragment corresponding to the CTD of the single-stranded SARS-CoV-2 RNA genome, showing micromolar affinity, and this fragment is surrounded by the basic grooves of the CTD [17, 28]. In addition, it has been demonstrated that CTD can self-bind to form oligomers (dimers, trimers, tetramers, or even octamers), and the instantaneous interaction between CTD dimers leads to the formation of higher-order oligomers. The degree of aggregation depends on the protein concentration [17, 72, 75, 77–80]. Similarly, the high-resolution crystal structure of CTD shows that it exists as a dimer in solution due to chain exchange resulting from close contact [28, 72]. The CTD detection and analysis of SARS-CoV-2 by static light scattering and chemical crosslinking showed that the SARS-CoV-2 CTD dimer is stable in solution, and the self-binding of this domain plays an important role in the overall N stability of SARS-CoV-2 [17, 81]. Most importantly, it was found that the C-terminal domain could also self-assemble and further mediate formation of the N protein tetramer [72, 82]. In addition, the SARS-CoV-2 CTD is crucial for liquid–liquid phase separation (LLPS) and NF-κB regulation of the N protein [83]. The formation mechanism contributing to LLPS is described in detail below.

### Structure and function of LKR and arms of the N protein

α-Helices and *β*-sheets are traditionally recognized as important elements of a protein’s secondary structure, and the intrinsically disordered regions (IDR) is becoming increasingly regarded as an important part of protein function [84–88]. The IDR lacks an inherent structure and is flexible and variable in form, which is why it can interact with biological macromolecules such as RNA, DNA, and proteins [85, 89, 90]. Previous studies have shown that there are three IDRs in the N proteins of SARS CoV-1, two of which are either at the N-terminal or the carboxyl terminal (N-arm and C-tail), whereas the third is in the central region (LKR) (Fig. 1 b) [64, 73, 84]. Similarly, there are also three IDRs in the N protein of SARS-CoV-2 [28, 72, 84, 87, 88]. The disordered C-tail of the N protein is thought to play a key role in the interaction with virus M protein and packaging signal [91]. The structural prediction of the C-tail of SARS-CoV-2 N protein shows that this region can form an instantaneous helix [87]. Cubuk et al. [82] reported formation of an instantaneous helix in the leucine-rich region using molecular dynamics simulation and suggested it provides an interface for oligomerization. Using structure prediction tools, Zhao et al. [87] confirmed the existence of a helix across residues 215–235 and found evidence of its role in protein oligomerization and co-assembly with N-terminal nucleic acid(NA) [87]. Regarding the conserved leucine-rich sequence 218–231, its potential role comes from its position in the junction region at 210–246, which has been found to be essential for RNA-mediated LLPS [87, 92]. In addition, hydrogen–deuterium exchange mass spectrometry analysis has shown that the conserved junction region rich in serine/arginine also has RNA-binding capacity [88]. At the N-terminal of the disordered linker, the region rich in SR is due to charged residues and groups serving as phosphorylation sites [87]. Their phosphorylation status is thought to regulate the function of the N protein through interactions of viral NSP3 protein with host proteins such as glycogen synthase 3, CDK-1, and 14–3-3 proteins [88, 93–96]. The R203K/G204R mutation has been experimentally shown to enhance the ability of the N protein to undergo agglutination, while R203M, which is commonly found in delta variants, can enhance virus replication [97, 98]. The most recently discovered mutation, G215C, is located in the linker between SR-rich and leucine-rich regions. G215C enhanced dimer–dimer interactions under reducing conditions and the possibility of forming disulfide bonds between different protoplasts [87]. In general, these regions are involved in interactions with viral RNA and proteins [21, 84, 87, 88, 99].

### Biological function of SARS-CoV-2 N protein

The N protein is the core component of the SARS-CoV-2 virus [28]. It is mainly responsible for identifying and wrapping the virus RNA into a helical symmetrical structure and plays an important multi-functional role in the life cycle of the coronavirus [21, 100, 101]. It binds to virus genomic RNA to form ribonucleoprotein complex (RNP). In addition to assembly, N proteins have other functions, including roles in viral mRNA transcription and replication, cytoskeletal tissue, and immune regulation [21, 28, 102–104]. In particular, N protein has been found to counteract host RNAi-mediated antiviral responses through its RNA binding activity, acting as a viral inhibitor through RNA silencing [56]. In addition, N protein can induce humoral and cellular immune response after infection, making it a key target for the development of diagnostics and vaccines [28, 105, 106]. N protein usually exists as a stable dimer [87]. Both NTD and CTD domains and connectors contribute to the binding of hybrid NA. The binding of NA induces a more ordered conformation, allowing dimer–dimer interactions, which in turn coordinate the N protein with scaffolding on NA, resulting in the co-assembly of polymers [87, 106]. Recent studies have shown that N proteins can be separated by liquid–liquid phase separation (LLPS) [101, 107–109]. NA binding also promotes LLPS, and the high concentration of N protein and NA co-condensates allows the formation of ribonucleoprotein particles [82, 92, 110]. The N protein also interacts with the SARS-CoV-2 membrane (M) protein, which seems to play a role in promoting N protein aggregates, fixing ribonucleoprotein particles on the virus membrane, and recognizing viral RNA [91, 92]. A recent study found that SARS-CoV-2 N protein binds to mannan-binding lectin (MBL)-associated serine protease 2 (masp2) and leads to complement overactivation and the aggravation of inflammatory lung injury [111, 112]. In addition, Oh and Shin [113] identified the role of the N protein in regulating antiviral immunity [113]. N protein overexpression leads to retinoic acid-induced gene-I (RIG-I)-like receptor-mediated interferon production and a reduction in interferon-induced gene expression. N protein inhibits the interaction between three-part motif protein 25 (TRIM25) and RIG-I [113]. In addition, N protein inhibits polyinosinic:polycytidylic-mediated interferon signal transduction at the level of Tank binding protein 1 (Tank-binding kinase1, TBK1), which interferes with the binding of TK1 to interferon regulatory factor 3 (IRF3), thus preventing nuclear translocation of IRF3 [113]. Another study showed that 11 s proteasome activator PA28γ can regulate intracellular abundance of the N protein [39]. Immunoprecipitation has been used to show that proteasome activator PA28*γ* is a nucleocapsid binding protein, whereby PA28γ binding plays an important role in regulating 20 s proteasome activity, which in turn regulates the level of SARS-CoV-2 key nucleocapsid protein [114].

### N protein post-translational modifications

**Fig. 4:**
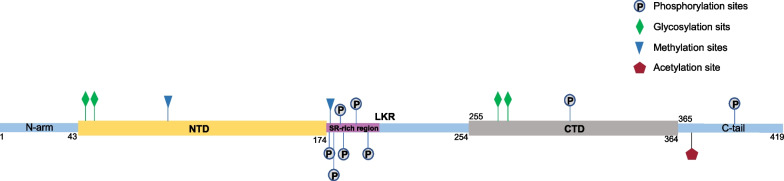
Domain structure and post-translational modification of the SARS-CoV-2 N protein

The N protein is post-translationally modified, and studying these modifications is very important for developing potential medical applications based on N protein [20]. Post-translational modifications of the N protein are shown in Fig. 4: First, phosphorylation plays an important role in regulating RNA binding and changing the physical and chemical properties of the N protein [88]. In the early stage of infection, the SR region is rapidly phosphorylated by cytoplasmic kinases at multiple sites [93, 95, 115, 116]. Phosphorylation leads to binding to RNA helicase DDX1, which promotes the structural RNA changes needed for long subgenomic RNA transcription in RTC [117]. Multivalent RNA–protein and protein–protein interactions based on unmodified N proteins lead to the formation partially ordered gel-like aggregates and discrete particles [93]. Phosphorylation of the C-terminal region disrupts these interactions, and the phosphorylated proteins form droplets that resemble liquids for viral genome processing [93]. In the late stage of infection, nucleocapsid formation and virus assembly do not seem to be dependent on N protein phosphorylation, which is significantly reduced in the nucleocapsids of MHV and SARS-CoV viruses [88, 93, 117]. N proteins form structured oligomers suitable for nucleocapsid assembly. Studies by Wu et al. [88] showed that mutations in S176, S188, and S206 in SR motifs lead to reduced RNA binding and a transition in protein–RNA populations with different solution properties. In addition, the highly phosphorylated state of the SR domain can regulate the interactions of the N protein with viral NSP3 proteins and host proteins such as glycogen synthase 3, CDK-1, and 14–3-3 proteins [20, 82, 87, 95, 115, 116, 118–123]. The phosphorylated N protein binds to the 14–3-3 protein in the host cytoplasm and regulates nucleoplasmic N protein shuttling [66, 96]. The phosphorylation of residues in the serine/arginine of LKR regulates discontinuous transcription, especially for shorter subgenomic mRNA that is closer to the 3' end in the early stage of replication [88, 99, 117].

Second, the N protein exhibits methylation modification. Cai et al. [124] demonstrated that the R95 and R177 residues in the RGG/RG motif of PRMT1-methylated N protein regulate the binding of N protein to its 5'-UTR genomic RNA. The methylation of R95 regulates the N protein by inhibiting the formation of stress granules (SGs). Arginine methylation affects nearby phosphorylation sites, which are usually antagonistic [124, 125]. The methylation of N proteins R95 and R177 is required for RNA binding [124]. Since S176, S180, S183, and S184 are phosphorylated by SRPK1 and GSK3 cell cycle-dependent kinase 1, there is likely to be an interaction between phosphorylation and methylation, especially in the vicinity of R177, in regulating binding to the 5'UTR of SARS-CoV-2 RNA [99, 117, 124, 126].

Finally, the N protein can undergo glycosylation and acetylation modification. Positions 48 and 270 of the N protein are N-glycosylation sites [127]. The Lys375 site is acetylated by host acetyltransferase, and acetylation-mimicking mutations occur frequently at this site, all of which adversely impact liquid–liquid separation of the P protein and RNA [128]. In addition, the post-translational modification of the N protein is also related to its production pathway [20]. The results of MS/MS and ^18^O labeling experiments showed that N47 and N269 of the N protein are modified by N-glycosylation when expressed in HEK293 cells. Phosphorylation of the T393 site was also observed. On the other hand, the natural N protein produced in the cell exhibits O-phosphorylation, at Ser176, but not glycosylation [20].

### Formation mechanism and function of liquid–liquid phase separation (LLPS)

Many RNA-binding proteins, especially those with a high proportion of inherently disordered regions, participate in liquid–liquid phase separation (LLPS) [97, 129–132]. The protein LLPS is a physical and chemical phenomenon that is considered to be the key mechanism for organizing macromolecules, such as proteins and nucleic acids, into membrane-free organelles [97, 133]. These membraneless cell compartments are dynamically assembled by LLPS and endow cells with the important ability to initiate biological functions or responses to a range of pressures [134–137]. After RNA virus infection, LLPS mediates the formation of stress granules and P-bodies. These substances play an important role in antiviral immunity by inhibiting the translation of viral mRNA and promoting RNA degradation [129–131]. LLPS is also considered to be the key to virus assembly [97, 138, 139]. A key step in coronavirus replication is the association of the N protein with virus genomic RNA, which then condenses into a higher-order RNA–protein complex, thus initiating the assembly of virions [97, 140]. To date, phase separation has been invoked or suggested in many virus environments [82, 141–144]. LLPS is involved in the interaction between the SARS-CoV-2 N protein–virus RNA complex and other viral proteins, such as nsp12 [82, 92, 101, 107–109]. Confocal fluorescence microscopy has shown that the N protein can easily self-bind into many micron-sized spherical aggregates, and its aggregates then fuse and condense into larger aggregates when reaching confluence, which serves as verification of the liquid characteristics of the N protein aggregates [101]. Fluorescence recovery after photobleaching was used to study the dynamics of internal molecules of N protein agglutinate [101]. It was shown that N proteins can diffuse partly freely in the condensed phase, which is consistent with their liquid behavior. In addition, the phase condensation of the N protein is very sensitive to increases in ionic strength, which indicates that the electrostatic interaction is very important for its condensation. The N protein can also experience LLPS in cells and exhibit liquid-like behavior [101]. Matsuo [30] summarized the formation mechanism of LLPS: RNA binding triggers LLPS of the N protein [97]. The N protein easily binds RNA, they are effectively co-separated at physiological salt concentration [101, 145, 146]. These droplets are formed by electrostatic interactions between positively charged N proteins and negatively charged RNA [30]. The shape of the droplet depends on the ratio of the N protein to RNA concentration [107, 109]. The size of the droplet is affected by the used RNA length [109]. The phase separation behavior also depends on the pH value and salt concentration [118]. and ATP can biphasically regulate LLPS by specifically binding to Arg/Lys residues in IDD, which is induced at low ATP concentrations but dissipates at high concentrations [118, 147, 148]. In addition, Each domain of SARS CoV-2 N contributes to its phase separation [101]. Nsp12 cannot undergo spontaneous separation, even if it is mixed with virus RNA, and it can be easily transformed into amorphous agglutinate with poor dynamic performance. However, nsp12 can be easily recruited into N protein–RNA aggregates without changing its shape or arrangement [101]. Similar results were obtained using RdRp complexes and ubiquitin-like domain 1 (Ubl1), which means that the N protein-driven LLPS may play an important role in the life cycle of SARS-CoV-2 [101, 102, 149].

LLPS also mediates the interaction between N protein/RNA and host. By binding to viral RNA, the N protein undergoes liquid–liquid phase separation and forms functional membraneless organelles to recruit TAK1 and IKK complexes, thus promoting the activation of NF-κB. Consistently, 1.6-hexanediol, an inhibitor of LLPS, attenuates SARS-CoV-2-induced NF-κB activation. LLPS of N protein/RNA contributes to virus-induced inflammation [83]. In cells, N protein forms aggregates and recruits stress granule protein G3BP1, highlighting the potential role of the N protein in isolating G3BP1 and inhibiting stress granules [92]. The phase separation of the N protein helps to inhibit G3BP1-dependent host immune response and package genomic RNA during virion assembly [92].

### Vaccine research and development

The N protein, spike protein, and membrane protein have been used in vaccine development due to their high immunogenicity [18, 30, 150–157]. Nucleocapsid-specific antibodies can improve protection against SARS-CoV-2 [158]. Gao et al. [159] used high-affinity glycan ligand-decorated glyconanoparticles to develop a universal SARS-CoV-2 vaccine (TCCSia-Ace-Dex-N-Rd vaccine), the TCCSia-Ace-Dex-N-Rd vaccine carries SARS-CoV-2 nucleocapsid protein (N) and can trigger strong N-specific CTL responses against target cells infected with SARS-CoV-2 and its variants of concern. Thura et al. [160] demonstrate novel vaccine candidates against SARS-CoV-2 by using the whole conserved N-protein or its fragment/peptides. The high titers of specific anti-N antibodies maintained for a reasonably long duration, suggesting that N-protein is a excellent immunogen to stimulate host immune system and enhance B-cells activation. Purified inactivated viruses manufactured by Sinovac and Sinopharm, such as CoronaVac and BBIBP-CorV, are options because they integrate not only S protein, but also other viral proteins, including matrix (M), envelope (E) and nucleocapsid (N) [161]. Studies have shown that in populations immunized with inactivated virus and the seropositivity rate is low, enhanced vaccinations significantly improves immunogenicity [162, 163]. Appelberg et al. [164] designed a universal SARS-CoV-2 DNA vaccine containing receptor-binding domain loops from the huCoV-19/WH01, the Alpha, and the Beta variants, combined with the membrane and nucleoproteins. The vaccine induced spike antibodies cross-reactive that neutralized huCoV-19/WH01, Beta, Delta, and Omicron virus in vitro, and primed nucleoprotein-specific T cells. Priming of cross-reactive nucleoprotein-specific T cells alone was 60% protective [164]. Similarly, other teams also designed and developed multiple recombinant vaccines based on nucleoproteins [165–168].The results showed that recombinant vaccines could significantly increase the levels of serum neutralizing antibody and total immunoglobulin [165], and induce strong specific lymphocyte proliferative response and T cell response [165]. The antigen specificity level of interferon-γ in splenocytes of immunized mice increased [166, 167]. The recently published reviews provided a good summary of the role of SARS-CoV-2 nucleocapsid protein in antiviral immunity and vaccine development [169, 170].

In addition, the N protein gene has attracted much attention as a potential drug target because it is more conservative, is more stable, and has fewer mutations than other viral proteins, such as spike proteins [171]. The drug research and development of the nucleocapsid protein is mainly based on its structure, function, and life cycle [30]. Since the RNA-binding activity of the N protein is very important for viral RNP formation and genome replication, the development of drugs that block RNA binding of the NTD or CTD has been proven to be a good antiviral strategy [28]. In recent studies, molecular docking and molecular dynamics simulation were used to identify potential antiviral drugs and to study the stability of NTD drug complexes [30, 172]. New strategies for the use of old drugs can be used in the treatment of new outbreaks of disease in a very short time and at a low cost. As a result, 34 drugs that have been approved or are under development were studied [30]. The results showed that rapamycin had the best binding affinity for NTD, and other compounds, such as saracatinib, camostat, trimetini and nafamostat, also showed high binding affinity and high stability [30]. Another study focused on compounds from medicinal plants. Five compounds were successfully isolated from 100 plant compounds: aloe-emodin, anthrarine, alizarine, dantron, and emodin [173–176]. Previous studies have shown that the compound PJ34, which targets the ribonucleotide binding site in NTD, can effectively inhibit the RNA-binding activity of the HCoV-OC43 N protein and inhibit viral replication [149]. By comparing the binding sites of PJ34 in the SARS-CoV-2 NTD structure with those in the HCoV-OC43 NTD structure, it is found that the key residues involved in the interaction are conserved [28, 149]. Dhankhar et al. [177] identified three small molecules with a conformation similar to guanosine monophosphate (GMP) at the active site of the NTD, and they exhibited high binding affinity and stable binding through the formation of hydrogen bonds with Arg107, Tyr111, and Arg149 of the N-terminal domain.

Second, we should consider screening inhibitors that block normal N protein oligomerization so as to prevent RNP formation or to induce abnormal aggregation. Recently, a new inhibitor, 5-benzyloxy Grammer (P3), was discovered by virtual screening [178]. This compound can mediate the NTD nonnative dimerization of MERS-CoV and induce N protein aggregation. It has been proven to have strong antiviral activity against MERS-CoV. By comparing the binding cavity of P3 with the corresponding parts of the SARS CoV-2 N-NTD structure, it was found that almost all the residues involved in the interaction are conserved [28, 178].

Third, many conserved coronavirus proteins, especially N proteins, need to be phosphorylated to be fully functional. Accordingly, Yaron et al. [179] identified alectinib through screening, a kinase inhibitor approved by the FDA that can inhibit N protein phosphorylation of SRPK1/2 and restrict SARS-CoV-2 replication. It is reported that the phosphorylated N protein dimer directly binds to the dimer 14–3-3 protein in a phosphorylation-dependent manner. The relatively tight 14–3-3/N protein binding can regulate nucleocytoplasmic shuttling and other functions of the N protein by blocking the SR enrichment region and hijacking the cell pathway by 14–3-3 protein isolation [96]. Therefore, this component may be a valuable target for therapeutic intervention. GSK-3 inhibitors block N protein phosphorylation and reduce the accumulation of viral RNA in cells. Targeting GSK-3 may provide a new strategy for addressing COVID-19 [96, 123].

The final antiviral therapy strategy involves indirect targeting of N protein regulation by inhibiting host cell kinases [180]. The site-specific phosphorylation of the SR domain by the host cell kinase seems to indicate that the N protein hijacks the host cell kinase for spatiotemporal regulation during the virus life cycle. SRPK1 seems to play an important role in the viral replication of many different viruses, as the inhibition or activation of SRPK1 may be beneficial to viruses [180–183]. The inhibition of SRPK1 and overactivation of SRPK1 may be effective antiviral strategies alone [182]. In addition, in cell culture models, the inhibition of SRPK1/2 has been shown to inhibit virus replication [182–184]. Similarly, viral replication SRPK1/2 inhibitors that have been shown to reduce SAR-SCoV-2 continue to be developed as potential cancer drugs, e.g., feasible strategies to interfere with SARS-CoV-2 replication and transmission include use of the FDA-approved kinase inhibitor alectinib, which strongly reacts with SRPK1 and inhibits SRPK1; the reuse of SRPK1/2 or GSK-3 inhibitors; or the development of new inhibitors [180, 185].

Furthermore, the formation and regulation of biomolecule condensates may be an important activity of the N protein that is essential for SARS-CoV-2 [180]. Cascarina and Ross [180] suggested that the N protein may use its ability to form or bind biomolecule condensates to disrupt stress particles, enhance viral replication or viral protein translation, and package the viral RNA genome into new virions. Considering the important role of the N protein in many stages of the virus life cycle, the regulation of the N protein through treatment with host cell kinase or nonmembrane organelle may be a feasible strategy to combat existing SARSCoV-2 infection [180].

### Diagnostic technology development

In addition to being a potential therapeutic or vaccine target, the N protein can also be used as an important diagnostic marker of COVID-19 [20, 24, 25, 186]. The presence of N protein peptides in gargles and nasopharyngeal swabs can be used for the immediate high-throughput detection of SARS-CoV-2 [20]. Fabiani et al. [187] used electrochemical immunosensors to detect SARS-CoV-2 S and N proteins in saliva at concentrations as low as 19 and 8 ng/mL, respectively. Cai et al. [11] developed an ultra-sensitive, rapid, and double digital enzyme-linked immunosorbent assay (dELISA) based on a single-molecule array, which is used to simulate the detection of the spike protein (S-RBD) and N protein. It shows supersensitivity and a high signal-to-noise ratio, which are helpful to improve the accuracy of COVID-19 diagnosis [11]. The detection of SARS-CoV-2 peptides through tandem mass spectrometry can be used as an alternative to polymerase chain reaction (PCR) and immunodiagnosis [20]. Clinical studies have shown that the peptide ^41^RPQGLPNNTASWFTALTQHGK^61^ in the N protein can be detected in the saliva of patients with COVID-19 [24]. Another study showed that strong signals for the N protein peptides ^375^ADETQALPQR^385^ and ^170^GYAQGSR^177^ can be rapidly detected in nasopharyngeal samples of patients with COVID-19 [20, 25].

This detection method based on the N protein serum level is helpful to accurately distinguish PCR-positive patients with COVID-19 from healthy and uninfected individuals [188–196]. Li et al. [188, 197] detected the N protein in the serum of COVID-19 patients with a sensitivity of 92% and a specificity of 97%. In another study, S and N proteins were detected in the plasma of patients with COVID-19 at concentrations ranging from 8 to 20,000 pg/mL and 0.8 to 1700 pg/mL, respectively [198]. Tan et al. [199] developed a microfluidic chemiluminescence ELISA platform that can detect S and N proteins in tenfold diluted serum within 40 min. Haljasmägi et al. [189] developed sensitive and specific LIPS methods for binding different SARS-CoV-2 antigens. The LIPS detection of S and N antigen fragments may provide useful information for the immune response of COVID-19 patients with different clinical courses [189, 190, 193]. Torrente-Rodriguez et al. [200] reported a multiplex electrochemical immunoassay for the detection of the N protein and S protein IgG and IgM in 100 × diluted serum samples. These studies have shown that the quantitative measurement of SARS-CoV-2 antigens, such as N and S proteins in serum/plasma, can be used for the accurate early detection of COVID-19 [188, 201].

## Conclusion

The outbreak of COVID-19 at the end of 2019 has had a wide range of medical, social, political, and financial implications. The high morbidity and mortality rate of COVID-19 has far exceeded that of seasonal influenza and other diseases. In order to curb the rapid spread of SARS-CoV-2 around the world, people have made great efforts to discover effective methods for diagnosis and treatment. Therefore, a detailed understanding of molecular events in the life cycle of SARS-CoV-2 and their underlying mechanisms, including virus replication and assembly, is urgently needed. Here, we summarize the progress in the research on SARS-CoV-2 N protein. It is important to deepen and develop our understanding of the structure and function of SARS-CoV-2 N proteins, their role in the life cycle of the virus, and their potential in vaccine and drug development.

In addition, to date, the molecular mechanisms involved in the SARS-CoV-2 life cycle after invading cells are not fully understood. In particular, SARS-CoV-2 has been constantly mutating and evolving new adaptation mechanisms, such as the "escape mechanism". More in-depth research is needed to discern the details. New coronavirus vaccines and diagnostic techniques robust to virus mutation have been developed.

Finally, specific drugs and treatments for COVID-19 have not yet been popularized; accurate diagnosis and a series of prevention and control measures are still the most effective means to prevent disease spread. Therefore, everyone must be vigilant, on the one hand, by strengthening their physical fitness levels to improve their own immunity and, on the other hand, by gathering scientific knowledge of self-protection to prevent infection in addition to establishing a routine for COVID-19 disease treatment in the case of future infections.

## Data Availability

Not applicable.

## Abbreviations

COVID-19: Coronavirus disease 2019
ORFs: Open reading frames
nsps: Nonstructural proteins
N protein: Nucleocapsid protein
LLPS: Liquid–liquid phase separation
S proteins: Spike proteins
RNP: Ribonucleotide complex
HCoV: Human-infecting coronaviruses
SARS-CoV: Severe acute respiratory syndrome coronavirus
MERS-CoV: Middle east respiratory syndrome coronavirus
NTD: N-terminal domain
CTD: C-terminal domain
IDRs: Intrinsically disordered regions
RBD: Receptor binding domain
ACE2: Receptor angiotensin-converting enzyme 2
CatB: Cysteine protease cathepsin B
RNAi: RNA interference
RdRp: RNA-dependent RNA polymerase
RTC: Replication-transcriptional complex
ER: Endoplasmic reticulum
DMVs: Double-membrane vesicles
ERGIC: ER–Golgi intermediate compartment
LKR: Central linking region
NA: N-terminal nucleic acid
MBL: Mannan-binding lectin
RIG-I: Retinoic acid-induced gene-I
TRIM25: Three-part motif protein 25
TBK1: Tank-binding kinase1
IRF3: Interferon regulatory factor 3
Ubl1: Ubiquitin-like domain 1
dELISA: Digital enzyme-linked immunosorbent assay

## References

[1] Szekely J, Mongkolprasert J, Jeayodae N, Senorit C, Chaimuti P, Swangphon P (2022). Development, analytical, and clinical evaluation of rapid immunochromatographic antigen test for SARS-CoV-2 variants detection. Diagnostics (Basel).

[2] Suthar TR, Gaikwad ST, Suthar AD (2020). Severe acute respiratory syndrome coronavirus 2 (SARS-CoV-2) and coronavirus disease-2019 (COVID-19): a review. Int J Curr Microbiol Appl Sci.

[3] Ma W, Yang J, Fu H, Su C, Yu C, Wang Q (2019). Genomic perspectives on the emerging SARS-CoV-2 omicron variant. Genomics Proteomics Bioinform.

[4] Aleem A, Samad ABA, Slenker AK. Emerging variants of SARS-CoV-2 and novel therapeutics against coronavirus (COVID-19). In: StatPearls. Treasure Island, StatPearls Publishing ; 2022.

[5] Mohapatra RK, Kandi V, Verma S, Dhama K (2022). Challenges of the Omicron (B.1.1.529) variant and its lineages: a global perspective. ChemBioChem.

[6] Tompa DR, Immanuel A, Srikanth S, Kadhirvel S (2021). Trends and strategies to combat viral infections: a review on FDA approved antiviral drugs. Int J Biol Macromol.

[7] El-Shabasy RM, Nayel MA, Taher MM, Abdelmonem R, Shoueir KR, Kenawy ER (2022). Three waves changes, new variant strains, and vaccination effect against COVID-19 pandemic. Int J Biol Macromol.

[8] Hassan SS, Lundstrom K, Barh D, Silva RJS, Andrade BS, Azevedo V (2021). Implications derived from S-protein variants of SARS-CoV-2 from six continents. Int J Biol Macromol.

[9] Hassan SS, Lundstrom K, Serrano-Aroca Á, Adadi P, Aljabali A, Redwan E (2022). Emergence of unique SARS-CoV-2 ORF10 variants and their impact on protein structure and function. Int J Biol Macromol.

[10] Souza PFN, Mesquita FP, Amaral JL, Landim PGC, Lima KRP, Costa MB (2022). The spike glycoprotein of SARS-CoV-2: a review of how mutations of spike glycoproteins have driven the emergence of variants with high transmissibility and immune escape. Int J Biol Macromol.

[11] Cai Q, Mu J, Lei Y, Ge J, Aryee AA, Zhang X (2021). Simultaneous detection of the spike and nucleocapsid proteins from SARS-CoV-2 based on ultrasensitive single molecule assays. Anal Bioanal Chem.

[12] Zheng J (2020). SARS-CoV-2: an emerging coronavirus that causes a global threat. Int J Biol Sci.

[13] Awadasseid A, Wu Y, Tanaka Y, Zhang W (2021). Current advances in the development of SARS-CoV-2 vaccines. Int J Biol Sci.

[14] Krammer F (2020). SARS-CoV-2 vaccines in development. Nature.

[15] Li C, Zhan W, Yang Z, Tu C, Hu G, Zhang X (2022). Broad neutralization of SARS-CoV-2 variants by an inhalable bispecific single-domain antibody. Cell.

[16] Altmann DM, Boyton RJ (2022). COVID-19 vaccination: the road ahead. Science.

[17] Zinzula L, Basquin J, Bohn S, Beck F, Klumpe S, Pfeifer G (2021). High-resolution structure and biophysical characterization of the nucleocapsid phosphoprotein dimerization domain from the Covid-19 severe acute respiratory syndrome coronavirus 2. Biochem Biophys Res Commun.

[18] Ahammad I, Lira SS (2020). Designing a novel mRNA vaccine against SARS-CoV-2: an immunoinformatics approach. Int J Biol Macromol.

[19] Dong Y, Dai T, Wei Y, Zhang L, Zheng M, Zhou F (2020). A systematic review of SARS-CoV-2 vaccine candidates. Sig Transduct Target Ther..

[20] Supekar NT, Shajahan A, Gleinich AS, Rouhani DS, Heiss C, Chapla DG (2021). Variable posttranslational modifications of severe acute respiratory syndrome coronavirus 2 nucleocapsid protein. Glycobiology.

[21] Chang CK, Hou MH, Chang CF, Hsiao CD, Huang TH (2014). The SARS coronavirus nucleocapsid protein–forms and functions. Antiviral Res.

[22] Burbelo PD, Riedo FX, Morishima C, Rawlings S, Smith D, Das S, et al. Detection of nucleocapsid antibody to SARS-CoV-2 is more sensitive than antibody to spike protein in COVID-19 patients. medRxiv 2020; 10.1101/2020.04.20.20071423.

[23] Yasui F, Kai C, Kitabatake M, Inoue S, Yoneda M, Yokochi S (2008). Prior immunization with severe acute respiratory syndrome (SARS)-associated coronavirus (SARS-CoV) nucleocapsid protein causes severe pneumonia in mice infected with SARS-CoV. J Immunol.

[24] Ihling C, Tänzler D, Hagemann S, Kehlen A, Hüttelmaier S, Arlt C (2020). Mass spectrometric identification of SARS-CoV-2 proteins from gargle solution samples of COVID-19 patients. J Proteome Res.

[25] Gouveia D, Miotello G, Gallais F, Gaillard JC, Debroas S, Bellanger L (2020). Proteotyping SARS-CoV-2 virus from nasopharyngeal swabs: a proof-of-concept focused on a 3 min mass spectrometry window. J Proteome Res.

[26] Baggen J, Vanstreels E, Jansen S, Daelemans D (2021). Cellular host factors for SARS-CoV-2 infection. Nat Microbiol.

[27] Yao H, Song Y, Chen Y, Wu N, Xu J, Sun C (2020). Molecular architecture of the SARS-CoV-2 virus. Cell.

[28] Peng Y, Du N, Lei Y, Dorje S, Qi J, Luo T (2020). Structures of the SARS-CoV-2 nucleocapsid and their perspectives for drug design. EMBO J.

[29] Michel CJ, Mayer C, Poch O, Thompson JD (2020). Characterization of accessory genes in coronavirus genomes. Virol J.

[30] Matsuo T (2021). Viewing SARS-CoV-2 nucleocapsid protein in terms of molecular flexibility. Biology.

[31] Bianchi M, Borsetti A, Ciccozzi M, Pascarella S (2021). SARS-Cov-2 ORF3a: mutability and function. Int J Biol Macromol.

[32] Hassan SS, Attrish D, Ghosh S, Choudhury PP, Uversky VN, Uhal BD (2020). Notable sequence homology of the ORF10 protein introspects the architecture of SARS-COV-2. Int J Biol Macromol.

[33] Yan L, Ge J, Zheng L, Zhang Y, Gao Y, Wang T (2021). Cryo-EM structure of an extended SARS-CoV-2 replication and transcription complex reveals an intermediate state in cap synthesis. Cell.

[34] Jin Z, Du X, Xu Y, Deng Y, Liu M, Zhao Y (2020). Structure of Mpro from SARS-CoV-2 and discovery of its inhibitors. Nature.

[35] Gao X, Qin B, Chen P, Zhu K, Hou P, Wojdyla JA (2021). Crystal structure of SARS-CoV-2 papain-like protease. Acta Pharm Sin B.

[36] Gao Y, Yan L, Huang Y, Liu F, Zhao Y, Cao L (2020). Structure of the RNA-dependent RNA polymerase from COVID-19 virus. Science.

[37] Walls AC, Park YJ, Tortorici MA, Wall A, McGuire AT, Veesler D (2020). Structure, function, and antigenicity of the SARS-CoV-2 spike glycoprotein. Cell.

[38] Kang S, Yang M, Hong Z, Zhang L, Huang Z, Chen X (2020). Crystal structure of SARS-CoV-2 nucleocapsid protein RNA binding domain reveals potential unique drug targeting sites. Acta Pharm Sin B.

[39] V'Kovski P, Kratzel A, Steiner S, Stalder H, Thiel V (2021). Coronavirus biology and replication: implications for SARS-CoV-2. Nat Rev Microbiol.

[40] Bouback TA, Samad A, Nur SM, Abdullah-Al-Mamun M, Alam R, Hossen MS (2021). Spike protein recognizer receptor ACE2 targeted identification of potential natural antiviral drug candidates against SARS-CoV-2. Int J Biol Macromol.

[41] Souza PFN, Lopes FES, Amaral JL, Freitas CDT, Oliveira JTA (2020). A molecular docking study revealed that synthetic peptides induced conformational changes in the structure of SARS-CoV-2 spike glycoprotein, disrupting the interaction with human ACE2 receptor. Int J Biol Macromol.

[42] Letko M, Marzi A, Munster V (2020). Functional assessment of cell entry and receptor usage for SARS-CoV-2 and other lineage B betacoronaviruses. Nat Microbiol.

[43] Hoffmann M, Kleine-Weber H, Schroeder S, Krüger N, Herrler T, Erichsen S (2020). SARS-CoV-2 cell entry depends on ACE2 and TMPRSS2 and is blocked by a clinically proven protease inhibitor. Cell.

[44] Lan J, Ge J, Yu J, Shan S, Zhou H, Fan S (2020). Structure of the SARS-CoV-2 spike receptor-binding domain bound to the ACE2 receptor. Nature.

[45] Shang J, Ye G, Shi K, Wan Y, Luo C, Aihara H (2020). Structural basis of receptor recognition by SARS-CoV-2. Nature.

[46] Shang J, Wan Y, Luo C, Ye G, Geng Q, Auerbach A (2020). Cell entry mechanisms of SARS-CoV-2. Proc Natl Acad Sci U S A.

[47] Gierer S, Bertram S, Kaup F, Wrensch F, Heurich A, Krämer-Kühl A (2013). The spike protein of the emerging betacoronavirus EMC uses a novel coronavirus receptor for entry, can be activated by TMPRSS2, and is targeted by neutralizing antibodies. J Virol.

[48] Matsuyama S, Nagata N, Shirato K, Kawase M, Takeda M, Taguchi F (2010). Efficient activation of the severe acute respiratory syndrome coronavirus spike protein by the transmembrane protease TMPRSS2. J Virol.

[49] Tortorici MA, Veesler D (2019). Structural insights into coronavirus entry. Adv Virus Res.

[50] Ou X, Liu Y, Lei X, Li P, Mi D, Ren L (2021). Characterization of spike glycoprotein of SARS-CoV-2 on virus entry and its immune cross-reactivity with SARS-CoV. Nat Commun.

[51] Yamamoto M, Matsuyama S, Li X, Takeda M, Kawaguchi Y, Inoue J-I (2016). Identification of Nafamostat as a potent inhibitor of middle east respiratory syndrome coronavirus s protein-mediated membrane fusion using the split-protein-based cell-cell fusion assay. Antimicrob Agents Chemother.

[52] Hoffmann M, Schroeder S, Kleine-Weber H, Müller MA, Drosten C, Pöhlmann S (2020). Nafamostat mesylate blocks activation of SARS-CoV-2: new treatment option for COVID-19. Antimicrob Agents Chemother.

[53] Menachery VD, Dinnon KH, Yount BL, McAnarney ET, Gralinski LE, Hale A (2020). Trypsin treatment unlocks barrier for zoonotic bat coronavirus infection. J Virol.

[54] Coutard B, Valle C, de Lamballerie X, Canard B, Seidah NG, Decroly E (2020). The spike glycoprotein of the new coronavirus 2019-nCoV contains a furin-like cleavage site absent in CoV of the same clade. Antiviral Res.

[55] Hoffmann M, Kleine-Weber H, Pöhlmann S (2020). A multibasic cleavage site in the spike protein of SARS-CoV-2 is essential for infection of human lung cells. Mol Cell.

[56] Mu J, Xu J, Zhang L, Shu T, Wu D, Huang M (2020). SARS-CoV-2-encoded nucleocapsid protein acts as a viral suppressor of RNA interference in cells. Sci China Life Sci.

[57] Setten RL, Rossi JJ, Han SP (2019). The current state and future directions of RNAi-based therapeutics. Nat Rev Drug Discov.

[58] Xin Y, Huang M, Guo WW, Huang Q, Zhang LZ, Jiang G (2017). Nano-based delivery of RNAi in cancer therapy. Mol Cancer.

[59] Perlman S, Netland J (2009). Coronaviruses post-SARS: update on replication and pathogenesis. Nat Rev Microbiol.

[60] Li F (2016). Structure, function, and evolution of coronavirus spike proteins. Annu Rev Virol.

[61] Ugurel OM, Mutlu O, Sariyer E, Kocer S, Ugurel E, Inci TG (2020). Evaluation of the potency of FDA-approved drugs on wild type and mutant SARS-CoV-2 helicase (Nsp13). Int J Biol Macromol.

[62] Khan MT, Irfan M, Ahsan H, Ahmed A, Kaushik AC, Khan AS (2021). Structures of SARS-CoV-2 RNA-binding proteins and therapeutic targets. Intervirology.

[63] Ghosh S, Dellibovi-Ragheb TA, Pak E, Qiu Q, Fisher M, Takvorian PM, et al. *β*-Coronaviruses use lysosomal organelles for cellular egress. biorxiv-192310. 2020.

[64] McBride R, van Zyl M, Fielding BC (2014). The coronavirus nucleocapsid is a multifunctional protein. Viruses.

[65] de Haan CA, Kuo L, Masters PS, Vennema H, Rottier PJ (1998). Coronavirus particle assembly: primary structure requirements of the membrane protein. J Virol.

[66] Surjit M, Kumar R, Mishra RN, Reddy MK, Chow VTK, Lal SK (2005). The severe acute respiratory syndrome coronavirus nucleocapsid protein is phosphorylated and localizes in the cytoplasm by 14-3-3-mediated translocation. J Virol.

[67] Kwarteng A, Asiedu E, Sakyi SA, Asiedu SO (2020). Targeting the SARS-CoV2 nucleocapsid protein for potential therapeutics using immuno-informatics and structure-based drug discovery techniques. Biomed Pharmacother.

[68] Surjit M, Lal SK (2008). The SARS-CoV nucleocapsid protein: a protein with multifarious activities. Infect Genet Evol.

[69] Schiavina M, Pontoriero L, Uversky VN, Felli IC, Pierattelli R (2021). The highly flexible disordered regions of the SARS-CoV-2 nucleocapsid N protein within the 1–248 residue construct: sequence-specific resonance assignments through NMR. Biomol NMR Assign.

[70] Peng TY, Lee KR, Tarn WY (2008). Phosphorylation of the arginine/serine dipeptide-rich motif of the severe acute respiratory syndrome coronavirus nucleocapsid protein modulates its multimerization, translation inhibitory activity and cellular localization. FEBS J.

[71] Dinesh DC, Chalupska D, Silhan J, Koutna E, Nencka R, Veverka V (2020). Structural basis of RNA recognition by the SARS-CoV-2 nucleocapsid phosphoprotein. PLoS Pathog.

[72] Bai Z, Cao Y, Liu W, Li J (2021). The SARS-CoV-2 nucleocapsid protein and its role in viral structure, biological functions, and a potential target for drug or vaccine mitigation. Viruses.

[73] Chang CK, Hsu YL, Chang YH, Chao FA, Wu MC, Huang YS (2009). Multiple nucleic acid binding sites and intrinsic disorder of severe acute respiratory syndrome coronavirus nucleocapsid protein: implications for ribonucleocapsid protein packaging. J Virol.

[74] Zhou R, Zeng R, von Brunn A, Lei J (2020). Structural characterization of the C-terminal domain of SARS-CoV-2 nucleocapsid protein. Mol Biomed.

[75] Chen CY, Chang CK, Chang YW, Sue SC, Bai HI, Riang L (2007). Structure of the SARS coronavirus nucleocapsid protein RNA-binding dimerization domain suggests a mechanism for helical packaging of viral RNA. J Mol Biol.

[76] Takeda M, Chang CK, Ikeya T, Güntert P, Chang Y-H, Hsu Y-L (2008). Solution structure of the c-terminal dimerization domain of SARS coronavirus nucleocapsid protein solved by the SAIL-NMR method. J Mol Biol.

[77] Chang CK, Chen CM, Chiang MH, Hsu YL, Huang TH (2013). Transient oligomerization of the SARS-CoV N protein–implication for virus ribonucleoprotein packaging. PLoS ONE.

[78] Luo H, Chen J, Chen K, Shen X, Jiang H (2006). Carboxyl terminus of severe acute respiratory syndrome coronavirus nucleocapsid protein: self-association analysis and nucleic acid binding characterization. Biochemistry.

[79] Surjit M, Liu B, Kumar P, Chow VTK, Lal SK (2004). The nucleocapsid protein of the SARS coronavirus is capable of self-association through a C-terminal 209 amino acid interaction domain. Biochem Biophys Res Commun.

[80] Yu IM, Gustafson CLT, Diao J, Burgner JW, Li Z, Zhang J (2005). Recombinant severe acute respiratory syndrome (SARS) coronavirus nucleocapsid protein forms a dimer through its C-terminal domain. J Biol Chem.

[81] Zeng W, Liu G, Ma H, Zhao D, Yang Y, Liu M (2020). Biochemical characterization of SARS-CoV-2 nucleocapsid protein. Biochem Biophys Res Commun.

[82] Cubuk J, Alston JJ, Incicco JJ, Singh S, Stuchell-Brereton MD, Ward MD (2021). The SARS-CoV-2 nucleocapsid protein is dynamic, disordered, and phase separates with RNA. Nat Commun.

[83] Wu Y, Ma L, Cai S, Zhuang Z, Zhao Z, Jin S (2021). RNA-induced liquid phase separation of SARS-CoV-2 nucleocapsid protein facilitates NF-κB hyper-activation and inflammation. Signal Transduct Target Ther.

[84] Barik S (2020). Genus-specific pattern of intrinsically disordered central regions in the nucleocapsid protein of coronaviruses. Comput Struct Biotechnol J.

[85] Dyson HJ, Wright PE (2005). Intrinsically unstructured proteins and their functions. Nat Rev Mol Cell Biol.

[86] Uversky VN, Dunker AK (1804). Understanding protein non-folding. Biochim Biophys Acta.

[87] Zhao H, Nguyen A, Wu D, Li Y, Hassan SA, Chen J, et al. Plasticity in structure and assembly of SARS-CoV-2 nucleocapsid protein. bioRxiv 2022; 10.1101/2022.02.08.479556. 10.1093/pnasnexus/pgac049 PMC9235412 35783502

[88] Wu C, Qavi AJ, Hachim A, Kavian N, Cole AR, Moyle AB, Wagner ND, Sweeney-Gibbons J, Rohrs HW, Gross ML, Peiris JM (2021). Characterization of SARS-CoV-2 nucleocapsid protein reveals multiple functional consequences of the C-terminal domain. Iscience.

[89] Dunker AK, Silman I, Uversky VN, Sussman JL (2008). Function and structure of inherently disordered proteins. Curr Opin Struct Biol.

[90] Nishikawa K Natively unfolded proteins: an overview. Biophysics (Nagoya-shi). 2009; 5: 53–8. 10.2142/biophysics.5.53 PMC5036634 27857579

[91] Masters PS (2019). Coronavirus genomic RNA packaging. Virology.

[92] Lu S, Ye Q, Singh D, Cao Y, Diedrich JK, Yates JR (2021). The SARS-CoV-2 nucleocapsid phosphoprotein forms mutually exclusive condensates with RNA and the membrane-associated M protein. Nat Commun.

[93] Carlson CR, Asfaha JB, Ghent CM, Howard CJ, Hartooni N, Safari M (2020). Phosphoregulation of phase separation by the SARS-CoV-2 N protein suggests a biophysical basis for its dual functions. Mol Cell.

[94] Koetzner CA, Hurst-Hess KR, Kuo L, Masters PS (2022). Analysis of a crucial interaction between the coronavirus nucleocapsid protein and the major membrane-bound subunit of the viral replicase-transcriptase complex. Virology.

[95] Bouhaddou M, Memon D, Meyer B, White KM, Rezelj VV, Correa Marrero M (2020). The global phosphorylation landscape of SARS-CoV-2 infection. Cell.

[96] Tugaeva KV, Hawkins DEDP, Smith JLR, Bayfield OW, Ker D-S, Sysoev AA (2021). The mechanism of SARS-CoV-2 nucleocapsid protein recognition by the human 14-3-3 proteins. J Mol Biol.

[97] Zhao M, Yu Y, Sun L-M, Xing J-Q, Li T, Zhu Y (2021). GCG inhibits SARS-CoV-2 replication by disrupting the liquid phase condensation of its nucleocapsid protein. Nat Commun.

[98] Syed AM, Taha TY, Tabata T, Chen IP, Ciling A, Khalid MM (2021). Rapid assessment of SARS-CoV-2-evolved variants using virus-like particles. Science.

[99] Wu C-H, Yeh S-H, Tsay Y-G, Shieh Y-H, Kao C-L, Chen Y-S (2009). Glycogen synthase kinase-3 regulates the phosphorylation of severe acute respiratory syndrome coronavirus nucleocapsid protein and viral replication. J Biol Chem.

[100] Malik YA (2020). Properties of coronavirus and SARS-CoV-2. Malays J Pathol.

[101] Zhao D, Xu W, Zhang X, Wang X, Ge Y, Yuan E (2021). Understanding the phase separation characteristics of nucleocapsid protein provides a new therapeutic opportunity against SARS-CoV-2. Protein Cell.

[102] Cong Y, Ulasli M, Schepers H, Mauthe M, V'Kovski P, Kriegenburg F (2020). Nucleocapsid protein recruitment to replication-transcription complexes plays a crucial role in coronaviral life cycle. J Virol.

[103] Lu X, Pan JA, Tao J, Guo D (2011). SARS-CoV nucleocapsid protein antagonizes IFN-*β* response by targeting initial step of IFN-*β* induction pathway, and its C-terminal region is critical for the antagonism. Virus Genes.

[104] Surjit M, Liu B, Chow VTK, Lal SK (2006). The nucleocapsid protein of severe acute respiratory syndrome-coronavirus inhibits the activity of cyclin-cyclin-dependent kinase complex and blocks S phase progression in mammalian cells. J Biol Chem.

[105] Ni L, Ye F, Cheng M-L, Feng Y, Deng Y-Q, Zhao H (2020). Detection of SARS-CoV-2-specific humoral and cellular immunity in COVID-19 convalescent individuals. Immunity.

[106] Xiang F, Wang X, He X, Peng Z, Yang B, Zhang J (2020). Antibody detection and dynamic characteristics in patients with Coronavirus disease 2019. Clin Infect Dis.

[107] Savastano A, Ibáñez de Opakua A, Rankovic M, Zweckstetter M. Nucleocapsid protein of SARS-CoV-2 phase separates into RNA-rich polymerase-containing condensates. Nat Commun 2020;11(1): 6041. 10.1038/s41467-020-19843-1 PMC7699647 33247108

[108] Perdikari TM, Murthy AC, Ryan VH, Watters S, Naik MT, Fawzi NL. SARS-CoV-2 nucleocapsid protein undergoes liquid-liquid phase separation stimulated by RNA and partitions into phases of human ribonucleoproteins. bioRxiv. 2020; 10.1101/2020.06.09.141101.

[109] Iserman C, Roden C, Boerneke M, Sealfon R, McLaughlin G, Jungreis I, et al. Specific viral RNA drives the SARS CoV-2 nucleocapsid to phase separate. bioRxiv 2020; 10.1101/2020.06.11.147199.

[110] Iserman C, Roden CA, Boerneke MA, Sealfon RSG, McLaughlin GA, Jungreis I (2020). Genomic RNA elements drive phase separation of the SARS-CoV-2 nucleocapsid. Mol Cell.

[111] Gao T, Hu M, Zhang X, Li H, Zhu L, Liu H, et al Highly pathogenic coronavirus N protein aggravates lung injury by MASP-2-mediated complement over-activation. medRxiv. 2020; 10.1101/2020.03.29.20041962.

[112] Kang S, Yang M, He S, Wang Y, Chen X, Chen Y-Q (2021). A SARS-CoV-2 antibody curbs viral nucleocapsid protein-induced complement hyperactivation. Nat Commun.

[113] Oh SJ, Shin OS (2021). SARS-CoV-2 nucleocapsid protein targets RIG-I-like receptor pathways to inhibit the induction of interferon response. Cells.

[114] Zhang H, Tu J, Cao C, Yang T, Gao L (2020). Proteasome activator PA28γ-dependent degradation of coronavirus disease (COVID-19) nucleocapsid protein. Biochem Biophys Res Commun.

[115] Klann K, Bojkova D, Tascher G, Ciesek S, Münch C, Cinatl J (2020). Growth factor receptor signaling inhibition prevents SARS-CoV-2 replication. Mol Cell.

[116] Davidson AD, Williamson MK, Lewis S, Shoemark D, Carroll MW, Heesom KJ (2020). Characterisation of the transcriptome and proteome of SARS-CoV-2 reveals a cell passage induced in-frame deletion of the furin-like cleavage site from the spike glycoprotein. Genome Med.

[117] Wu C-H, Chen P-J, Yeh S-H (2014). Nucleocapsid phosphorylation and RNA helicase DDX1 recruitment enables coronavirus transition from discontinuous to continuous transcription. Cell Host Microbe.

[118] Perdikari TM, Murthy AC, Ryan VH, Watters S, Naik MT, Fawzi NL (2020). SARS-CoV-2 nucleocapsid protein phase-separates with RNA and with human hnRNPs. EMBO J.

[119] Catanzaro M, Fagiani F, Racchi M, Corsini E, Govoni S, Lanni C (2020). Immune response in COVID-19: addressing a pharmacological challenge by targeting pathways triggered by SARS-CoV-2. Signal Transduct Target Ther.

[120] Boehning M, Dugast-Darzacq C, Rankovic M, Hansen AS, Yu T, Marie-Nelly H (2018). RNA polymerase II clustering through carboxy-terminal domain phase separation. Nat Struct Mol Biol.

[121] Zheng Y, Zhuang M-W, Han L, Zhang J, Nan M-L, Zhan P (2020). Severe acute respiratory syndrome coronavirus 2 (SARS-CoV-2) membrane (M) protein inhibits type I and III interferon production by targeting RIG-I/MDA-5 signaling. Signal Transduct Target Ther.

[122] Chen K, Xiao F, Hu D, Ge W, Tian M, Wang W (2020). SARS-CoV-2 nucleocapsid protein interacts with RIG-I and represses RIG-mediated IFN-*β* production. Viruses.

[123] Liu X, Verma A, Garcia G, Ramage H, Myers RL, Lucas A, et al. Targeting the coronavirus nucleocapsid protein through GSK-3 inhibition. medRxiv 2021; 10.1101/2021.02.17.21251933. 10.1073/pnas.2113401118 PMC8594528 34593624

[124] Cai T, Yu Z, Wang Z, Liang C, Richard S (2021). Arginine methylation of SARS-Cov-2 nucleocapsid protein regulates RNA binding, its ability to suppress stress granule formation, and viral replication. J Biol Chem.

[125] Guccione E, Richard S (2019). The regulation, functions and clinical relevance of arginine methylation. Nat Rev Mol Cell Biol.

[126] Heaton BE, Trimarco JD, Hamele CE, Harding AT, Tata A, Zhu X, et al. SRSF protein kinases 1 and 2 are essential host factors for human coronaviruses including SARS-CoV-2. bioRxiv. 2020; 10.1101/2020.08.14.251207.

[127] Rump A, Risti R, Kristal M-L, Reut J, Syritski V, Lookene A (2021). Dual ELISA using SARS-CoV-2 nucleocapsid protein produced in *E. coli* and CHO cells reveals epitope masking by N-glycosylation. Biochem Biophys Res Commun.

[128] Wang S, Dai T, Qin Z, Pan T, Chu F, Lou L (2021). Targeting liquid–liquid phase separation of SARS-CoV-2 nucleocapsid protein promotes innate antiviral immunity by elevating MAVS activity. Nat Cell Biol.

[129] Sanders DW, Kedersha N, Lee DSW, Strom AR, Drake V, Riback JA (2020). Competing protein-RNA interaction networks control multiphase intracellular organization. Cell.

[130] Yang P, Mathieu C, Kolaitis R-M, Zhang P, Messing J, Yurtsever U (2020). G3BP1 is a tunable switch that triggers phase separation to assemble stress granules. Cell.

[131] Guillén-Boixet J, Kopach A, Holehouse AS, Wittmann S, Jahnel M, Schlüßler R (2020). RNA-induced conformational switching and clustering of G3BP drive stress granule assembly by condensation. Cell.

[132] Burke KA, Janke AM, Rhine CL, Fawzi NL (2015). Residue-by-residue view of in vitro FUS granules that bind the C-terminal domain of RNA polymerase II. Mol Cell.

[133] Boeynaems S, Alberti S, Fawzi NL, Mittag T, Polymenidou M, Rousseau F (2018). Protein phase separation: a new phase in cell biology. Trends Cell Biol.

[134] Franzmann TM, Alberti S (2019). Prion-like low-complexity sequences: key regulators of protein solubility and phase behavior. J Biol Chem.

[135] Riback JA, Katanski CD, Kear-Scott JL, Pilipenko EV, Rojek AE, Sosnick TR (2017). Stress-triggered phase separation is an adaptive, evolutionarily tuned response. Cell.

[136] Brangwynne CP, Eckmann CR, Courson DS, Rybarska A, Hoege C, Gharakhani J (2009). Germline P granules are liquid droplets that localize by controlled dissolution/condensation. Science.

[137] Shin Y, Brangwynne CP (2017). Liquid phase condensation in cell physiology and disease. Science.

[138] Jobe F, Simpson J, Hawes P, Guzman E, Bailey D (2020). Respiratory syncytial virus sequesters NF-κB subunit p65 to cytoplasmic inclusion bodies to inhibit innate immune signaling. J Virol.

[139] Guseva S, Milles S, Jensen MR, Salvi N, Kleman J-P, Maurin D (2020). Measles virus nucleo- and phosphoproteins form liquid-like phase-separated compartments that promote nucleocapsid assembly. Sci Adv.

[140] Vennema H, Godeke GJ, Rossen JW, Voorhout WF, Horzinek MC, Opstelten DJ (1996). Nucleocapsid-independent assembly of coronavirus-like particles by co-expression of viral envelope protein genes. EMBO J.

[141] Nikolic J, Le Bars R, Lama Z, Scrima N, Lagaudrière-Gesbert C, Gaudin Y (2017). Negri bodies are viral factories with properties of liquid organelles. Nat Commun.

[142] Metrick CM, Koenigsberg AL, Heldwein EE (2020). Conserved outer tegument component UL11 from Herpes simplex virus 1 is an intrinsically disordered. RNA-binding protein mBio.

[143] Monette A, Niu M, Chen L, Rao S, Gorelick RJ, Mouland AJ (2020). Pan-retroviral nucleocapsid-mediated phase separation regulates genomic RNA positioning and trafficking. Cell Rep.

[144] Monette A, Mouland AJ (2020). Zinc and copper ions differentially regulate prion-like phase separation dynamics of pan-virus nucleocapsid biomolecular condensates. Viruses.

[145] Mei D, Lim L, Kang J, Song J. ATP regulates TDP-43 pathogenesis by specifically binding to an inhibitory component of a delicate network controlling LLPS. biorxiv 2020; 10.1101/2020.10.08.330829.

[146] Nelson GW, Stohlman SA, Tahara SM (2000). High affinity interaction between nucleocapsid protein and leader/intergenic sequence of mouse hepatitis virus RNA. Microbiology.

[147] Dang M, Li Y, Song J (2021). ATP biphasically modulates LLPS of SARS-CoV-2 nucleocapsid protein and specifically binds its RNA-binding domain. Biochem Biophys Res Commun.

[148] Kang J, Lim L, Lu Y, Song J (2019). A unified mechanism for LLPS of ALS/FTLD-causing FUS as well as its modulation by ATP and oligonucleic acids. PLoS Biol.

[149] Lin S-Y, Liu C-L, Chang Y-M, Zhao J, Perlman S, Hou M-H (2014). Structural basis for the identification of the N-terminal domain of coronavirus nucleocapsid protein as an antiviral target. J Med Chem.

[150] Okada M, Takemoto Y, Okuno Y, Hashimoto S, Yoshida S, Fukunaga Y (2005). The development of vaccines against SARS corona virus in mice and SCID-PBL/hu mice. Vaccine.

[151] Gao W, Tamin A, Soloff A, D'Aiuto L, Nwanegbo E, Robbins PD (2003). Effects of a SARS-associated coronavirus vaccine in monkeys. Lancet.

[152] Collier DA, Ferreira IATM, Kotagiri P, Datir R, Lim E, Touzier E (2021). Age-related immune response heterogeneity to SARS-CoV-2 vaccine BNT162b2. Nature.

[153] Felipe LS, Vercruysse T, Sharma S, Ma J, Lemmens V, van Looveren D (2021). A single-dose live-attenuated YF17D-vectored SARS-CoV-2 vaccine candidate. Nature.

[154] Sternberg A, Naujokat C (2020). Structural features of coronavirus SARS-CoV-2 spike protein: targets for vaccination. Life Sci.

[155] Mohammed MEA (2022). SARS-CoV-2 proteins: are they useful as targets for COVID-19 drugs and vaccines?. Curr Mol Med.

[156] Fathizadeh H, Afshar S, Masoudi MR, Gholizadeh P, Asgharzadeh M, Ganbarov K (2021). SARS-CoV-2 (Covid-19) vaccines structure, mechanisms and effectiveness: a review. Int J Biol Macromol.

[157] Lundstrom K, Hromić-Jahjefendić A, Bilajac E, Aljabali AAA, Baralić K, Sabri NA, Shehata EM, Raslan M, Raslan SA, Ferreira A (2022). COVID-19 signalome: potential therapeutic interventions. Cell Signal.

[158] Dangi T, Sanchez S, Class J, Richner M, Visvabharathy L, Chung YR, Bentley K, Stanton RJ, Koralnik IJ, Richner JM, Penaloza-MacMaster P (2022). Improved control of SARS-CoV-2 by treatment with a nucleocapsid-specific monoclonal antibody. J Clin Invest.

[159] Gao Y, Wang W, Yang Y, Zhao Q, Yang C, Jia X, Liu Y, Zhou M, Zeng W, Huang X, et al: developing next-generation protein-based vaccines using high-affinity glycan ligand-decorated glyconanoparticles. Adv Sci. 2022;e2204598. 10.1002/advs.202204598 PMC9839878 36398611

[160] Thura M, Sng JXE, Ang KH, Li J, Gupta A, Hong JM, Hong CW, Zeng Q (2021). Targeting intra-viral conserved nucleocapsid (N) proteins as novel vaccines against SARS-CoVs. Biosci Rep.

[161] Dinc HO, Saltoglu N, Can G, Balkan II, Budak B, Ozbey D, Caglar B, Karaali R, Mete B, Tuyji Tok Y (2022). Inactive SARS-CoV-2 vaccine generates high antibody responses in healthcare workers with and without prior infection. Vaccine.

[162] Oronsky B, Larson C, Caroen S, Hedjran F, Sanchez A, Prokopenko E, Reid T (2022). Nucleocapsid as a next-generation COVID-19 vaccine candidate. Int J Infect Dis.

[163] Cheng H, Peng Z, Si S, Alifu X, Zhou H, Chi P, Zhuang Y, Mo M, Yu Y (2022). Immunogenicity and safety of homologous and heterologous prime-boost immunization with COVID-19 vaccine: systematic review and meta-analysis. Vaccines (Basel)..

[164] Appelberg S, Ahlén G, Yan J, Nikouyan N, Weber S, Larsson O, Höglund U, Aleman S, Weber F, Perlhamre E (2022). A universal SARS-CoV DNA vaccine inducing highly cross-reactive neutralizing antibodies and T cells. EMBO Mol Med.

[165] Ghaemi A, Roshani Asl P, Zargaran H, Ahmadi D, Hashimi AA, Abdolalipour E, Bathaeian S, Miri SM (2022). Recombinant COVID-19 vaccine based on recombinant RBD/Nucleoprotein and saponin adjuvant induces long-lasting neutralizing antibodies and cellular immunity. Front Immunol.

[166] Feng W, Xiang Y, Wu L, Chen Z, Li Q, Chen J, Guo Y, Xia D, Chen N, Zhang L (2022). Nucleocapsid protein of SARS-CoV-2 is a potential target for developing new generation of vaccine. J Clin Lab Anal.

[167] Özcengiz E, Keser D, Özcengiz G, Çelik G, Özkul A, İnçeh FN (2022). Two formulations of coronavirus disease-19 recombinant subunit vaccine candidate made up of S1 fragment protein P1, S2 fragment protein P2, and nucleocapsid protein elicit strong immunogenicity in mice. Immun Inflamm Dis.

[168] Lam JY, Ng YY, Yuen CK, Wong WM, Yuen KY, Kok KH (2022). A nasal omicron vaccine booster elicits potent neutralizing antibody response against emerging SARS-CoV-2 variants. Emerg Microbes Infect.

[169] Yu H, Guan F, Miller H, Lei J, Liu C (2022). The role of SARS-CoV-2 nucleocapsid protein in antiviral immunity and vaccine development. Emerg Microbes Infect.

[170] Zhang B, Tian J, Zhang Q, Xie Y, Wang K, Qiu S, Lu K, Liu Y (2022). Comparing the nucleocapsid proteins of human coronaviruses: structure, immunoregulation, vaccine, and targeted drug. Front Mol Biosci.

[171] Dutta NK, Mazumdar K, Gordy JT (2020). The nucleocapsid protein of SARS-CoV-2: a target for vaccine development. J Virol.

[172] Tatar G, Ozyurt E, Turhan K (2021). Computational drug repurposing study of the RNA binding domain of SARS-CoV-2 nucleocapsid protein with antiviral agents. Biotechnol Prog.

[173] Rolta R, Yadav R, Salaria D, Trivedi S, Imran M, Sourirajan A (2021). In silico screening of hundred phytocompounds of ten medicinal plants as potential inhibitors of nucleocapsid phosphoprotein of COVID-19: an approach to prevent virus assembly. J Biomol Struct Dyn.

[174] Sarma P, Shekhar N, Prajapat M, Avti P, Kaur H, Kumar S (2021). In-silico homology assisted identification of inhibitor of RNA binding against 2019-nCoV N-protein (N terminal domain). J Biomol Struct Dyn.

[175] Yadav R, Imran M, Dhamija P, Suchal K, Handu S (2021). Virtual screening and dynamics of potential inhibitors targeting RNA binding domain of nucleocapsid phosphoprotein from SARS-CoV-2. J Biomol Struct Dyn.

[176] Amin M, Abbas G (2020). Docking study of chloroquine and hydroxychloroquine interaction with RNA binding domain of nucleocapsid phospho-protein - an *in silico* insight into the comparative efficacy of repurposing antiviral drugs. J Biomol Struct Dyn.

[177] Dhankhar P, Dalal V, Singh V, Tomar S, Kumar P (2022). Computational guided identification of novel potent inhibitors of N-terminal domain of nucleocapsid protein of severe acute respiratory syndrome coronavirus 2. J Biomol Struct Dyn.

[178] Lin S-M, Lin S-C, Hsu J-N, Chang C-K, Chien C-M, Wang Y-S (2020). Structure-based stabilization of non-native protein-protein interactions of coronavirus nucleocapsid proteins in antiviral drug design. J Med Chem.

[179] Yaron TM, Heaton BE, Levy TM, Johnson JL, Jordan TX, Cohen BM, et al. The FDA-approved drug Alectinib compromises SARS-CoV-2 nucleocapsid phosphorylation and inhibits viral infection in vitro. bioRxiv. 2020; 10.1101/2020.08.14.251207.

[180] Cascarina SM, Ross ED (2020). A proposed role for the SARS-CoV-2 nucleocapsid protein in the formation and regulation of biomolecular condensates. FASEB J.

[181] Prescott EL, Brimacombe CL, Hartley M, Bell I, Graham S, Roberts S (2014). Human papillomavirus type 1 E1^E4 protein is a potent inhibitor of the serine-arginine (SR) protein kinase SRPK1 and inhibits phosphorylation of host SR proteins and of the viral transcription and replication regulator E2. J Virol.

[182] Takamatsu Y, Krähling V, Kolesnikova L, Halwe S, Lier C, Baumeister S, Noda T, Biedenkopf N, Becker S (2020). Serine-arginine protein kinase 1 regulates Ebola virus transcription. MBio.

[183] Fukuhara T, Hosoya T, Shimizu S, Sumi K, Oshiro T, Yoshinaka Y (2006). Utilization of host SR protein kinases and RNA-splicing machinery during viral replication. Proc Natl Acad Sci U S A.

[184] Karakama Y, Sakamoto N, Itsui Y, Nakagawa M, Tasaka-Fujita M, Nishimura-Sakurai Y (2010). Inhibition of hepatitis C virus replication by a specific inhibitor of serine-arginine-rich protein kinase. Antimicrob Agents Chemother.

[185] Hatcher JM, Wu G, Zeng C, Zhu J, Meng F, Patel S (2018). SRPKIN-1: a covalent SRPK1/2 inhibitor that potently converts VEGF from pro-angiogenic to anti-angiogenic isoform. Cell Chem Biol.

[186] Sharma A, Balda S, Apreja M, Kataria K, Capalash N, Sharma P (2021). COVID-19 diagnosis: current and future techniques. Int J Biol Macromol.

[187] Fabiani L, Saroglia M, Galatà G, De Santis R, Fillo S, Luca V (2021). Magnetic beads combined with carbon black-based screen-printed electrodes for COVID-19: a reliable and miniaturized electrochemical immunosensor for SARS-CoV-2 detection in saliva. Biosens Bioelectron.

[188] Li J, Lillehoj PB (2021). Microfluidic magneto immunosensor for rapid, high sensitivity measurements of SARS-CoV-2 nucleocapsid protein in serum. ACS Sens.

[189] Haljasmägi L, Remm A, Rumm AP, Krassohhina E, Sein H, Tamm A (2020). LIPS method for the detection of SARS-CoV-2 antibodies to spike and nucleocapsid proteins. Eur J Immunol.

[190] Burbelo PD, Riedo FX, Morishima C, Rawlings S, Smith D, Das S (2020). Sensitivity in detection of antibodies to nucleocapsid and spike proteins of severe acute respiratory syndrome coronavirus 2 in patients with coronavirus disease 2019. J Infect Dis.

[191] Burbelo PD, Ching KH, Klimavicz CM, Iadarola MJ (2009). Antibody profiling by luciferase immunoprecipitation systems (LIPS). J Vis Exp.

[192] To KK, Tsang OT, Leung WS, Tam AR, Wu TC, Lung DC, Yip CC, Cai JP, Chan JM, Chik TS, Lau DP (2020). Temporal profiles of viral load in posterior oropharyngeal saliva samples and serum antibody responses during infection by SARS-CoV-2: an observational cohort study. Lancet Infectious Diseases.

[193] Guo L, Ren L, Yang S, Xiao M, Chang D, Yang F (2020). Profiling early humoral response to diagnose novel coronavirus disease (COVID-19). Clin Infect Dis.

[194] Zhao J, Yuan Q, Wang H, Liu W, Liao X, Su Y (2020). Antibody responses to SARS-CoV-2 in patients with novel coronavirus disease 2019. Clin Infect Dis.

[195] Wölfel R, Corman VM, Guggemos W, Seilmaier M, Zange S, Müller MA (2020). Virological assessment of hospitalized patients with COVID-2019. Nature.

[196] Li Z, Yi Y, Luo X, Xiong N, Liu Y, Li S (2020). Development and clinical application of a rapid IgM-IgG combined antibody test for SARS-CoV-2 infection diagnosis. J Med Virol.

[197] Li T, Wang L, Wang H, Li X, Zhang S, Xu Y (2020). Serum SARS-COV-2 nucleocapsid protein: a sensitivity and specificity early diagnostic marker for SARS-COV-2 infection. Front Cell Infect Microbiol.

[198] Ogata AF, Maley AM, Wu C, Gilboa T, Norman M, Lazarovits R (2020). Ultra-sensitive serial profiling of SARS-CoV-2 antigens and antibodies in plasma to understand disease progression in COVID-19 patients with severe disease. Clin Chem.

[199] Tan X, Krel M, Dolgov E, Park S, Li X, Wu W (2020). Rapid and quantitative detection of SARS-CoV-2 specific IgG for convalescent serum evaluation. Biosens Bioelectron.

[200] Torrente-Rodríguez RM, Lukas H, Tu J, Min J, Yang Y, Xu C (2020). SARS-CoV-2 RapidPlex: a graphene-based multiplexed telemedicine platform for rapid and low-cost COVID-19 diagnosis and monitoring. Matter.

[201] Khan WH, Khan N, Mishra A, Gupta S, Bansode V, Mehta D (2022). Dimerization of SARS-CoV-2 nucleocapsid protein affects sensitivity of ELISA based diagnostics of COVID-19. Int J Biol Macromol.

